# Accuracy and Reliability of Automated Gray Matter Segmentation Pathways on Real and Simulated Structural Magnetic Resonance Images of the Human Brain

**DOI:** 10.1371/journal.pone.0045081

**Published:** 2012-09-18

**Authors:** Lucas D. Eggert, Jens Sommer, Andreas Jansen, Tilo Kircher, Carsten Konrad

**Affiliations:** 1 Institute of Cognitive Science, University of Osnabrück, Osnabrück, Germany; 2 Department of Psychiatry and Psychotherapy, Philipps-University Marburg, Marburg, Germany; Institution of Automation, CAS, China

## Abstract

Automated gray matter segmentation of magnetic resonance imaging data is essential for morphometric analyses of the brain, particularly when large sample sizes are investigated. However, although detection of small structural brain differences may fundamentally depend on the method used, both accuracy and reliability of different automated segmentation algorithms have rarely been compared. Here, performance of the segmentation algorithms provided by SPM8, VBM8, FSL and FreeSurfer was quantified on simulated and real magnetic resonance imaging data. First, accuracy was assessed by comparing segmentations of twenty simulated and 18 real T1 images with corresponding ground truth images. Second, reliability was determined in ten T1 images from the same subject and in ten T1 images of different subjects scanned twice. Third, the impact of preprocessing steps on segmentation accuracy was investigated. VBM8 showed a very high accuracy and a very high reliability. FSL achieved the highest accuracy but demonstrated poor reliability and FreeSurfer showed the lowest accuracy, but high reliability. An universally valid recommendation on how to implement morphometric analyses is not warranted due to the vast number of scanning and analysis parameters. However, our analysis suggests that researchers can optimize their individual processing procedures with respect to final segmentation quality and exemplifies adequate performance criteria.

## Introduction

Automated brain segmentation algorithms segment a structural magnetic resonance imaging (MRI) image into different tissue classes. In general, a MRI image is segmented into gray matter, white matter, and cerebrospinal fluid. Based on this segmentation, methods are available to calculate several neuroanatomical measures, for example gray matter volume, gray matter density, cortical thickness, or cortical curvature. Researchers use these measures to investigate differences in brain structure between groups or to investigate changes in brain structure over time. Phenomena that are investigated include learning processes [1], language lateralization [2], psychosis [3], mild cognitive impairment [4], [5], aphasia [6], alexithymia [7], post-traumatic stress disorder [8], Huntington disease [9], [10], depression [11], autism [12], [13], and schizophrenia [14]. The use of *automated* segmentation algorithms is desirable, as these algorithms are (i) much faster than manual segmentations and (ii) user independent, that is, they do not depend on expert knowledge in neuroanatomy. However, significant challenges exist as differences in brain structure between groups, or changes within subjects are often very subtle (please see, e.g., [15], [16]). Therefore, it is crucially important that (i) automated segmentation algorithms are able to precisely determine the exact amount of, for example, gray matter tissue in an MRI image (cf. accuracy), and that (ii) they produce similar results, when applied to different images of the same person (cf. reliability). At the moment, however, too little is known about the accuracy and reliability of current automated segmentation algorithms.

Clark et al. [17] addressed the problem of reliability of different automated segmentation algorithms. Combining different algorithms for intensity correction, skull-stripping and segmentation, Clark et al. [17] produced a large number of different processing pathways and tested these pathways on twenty MRI images taken from the same subject. They found that the most “optimal” processing pathway yielded volume estimates that were on average three times less variable than those estimates calculated by less “optimal” pathways. They also demonstrated that the choice of the segmentation algorithm had the greatest impact on the variability of the final segmentation, whereas intensity correction and skull-stripping algorithms had little effect on the overall tissue segmentation reliability. In contrast to those findings, Fein et al. [18] showed that skull-stripping may greatly improve the power of structural brain analysis. Acosta-Cabronero et al. [19] evaluated the impact of skull-stripping and intensity correction algorithms on the subsequent segmentation. In accordance with the findings of Fein et al. [18], they reported a large influence of those preprocessing steps.

In 2009, Klauschen et al. [20] conducted a systematic evaluation of different segmentation algorithms. They used simulated brain data that were generated based on varying brain anatomy and varying image quality, as well as real images from nine different individuals and test-retest images of 48 individuals. They tested the performance of three commonly used segmentation algorithms, provided by software packages SPM5, FSL, and FreeSurfer. Within-segmenter analyses revealed volume differences greater than 15%. Between-segmenter comparisons showed an average discrepancy of 24% for real MRI images. The results of Klauschen et al. [20] suggested that automated brain segmentation algorithms might be seriously limited in the fine discrimination of tissue classes. Most importantly, their study casted serious doubts on the capability of automated segmentation algorithms to detect changes in brain structure in longitudinal studies.

To provide information to the community regarding which gray matter segmentation procedure they can build upon, we present a systematic evaluation of *accuracy and reliability* of *standard* gray matter segmentation algorithms. Whereas Clark et al. [17] emphasized the comparison of different processing pipelines with permuting preprocessing steps, and whereas Klauschen et al. [20] tested within and between-segmenter reliability and accuracy of three software packages, our investigation expands the work by Clark et al. and Klauschen et al. by providing a comprehensive investigation of *both* segmentation pipelines and within and between-segmenter accuracy and reliability using the *latest versions* of commonly used segmentation algorithms. Importantly, we provide measures of accuracy obtained from real T1 MRI images. To our knowledge this has not been done before in a systematical manner. The fact that we tested the latest versions of available segmentations procedures is also of particular importance, because, up to now, all studies concerned with the evaluation of automated segmentation [17], [20] used segmentation algorithms that were subjected to substantial development since.

In the current study, we evaluated the segmentation algorithms provided by (i) SPM8, (ii) VBM8, (iii) FSL, and (iv) FreeSurfer separately and in combination with algorithms for intensity correction and skull-stripping. We determined *accuracy* in terms of the Dice coefficient computed for the comparison of ground truth images and corresponding gray matter segmentations in simulated and real T1 brain images. We evaluated *reliability* in terms of standard deviation, coefficient of variation, and reliability coefficient of gray matter segmentations on real T1 images. In comparison to previous studies, our focus was on the simultaneous investigation of accuracy and reliability in combination with a systematic evaluation of the influence of each processing step on segmentation quality. Thus, we were able to examine (a) which processing step has the largest influence on segmentation accuracy *both in simulated and real* T1 MRI images, (b) how accuracy and reliability are linked, (c) how results from simulated and real T1 images differ, and (d) how preprocessing steps and segmentation algorithms interact.

## Materials and Methods

### Data Sets

To investigate the accuracy of different segmentation pathways we used (i) twenty simulated T1-weighted MRI images and corresponding discrete anatomical models provided by the Simulated Brain Database (http://mouldy.bic.mni.mcgill.ca/brainweb/; “BrainWeb data set”) and (ii) 18 real T1-weighted MRI images with expert segmentations of 43 individual structures from the Internet Brain Segmentation Repository (“IBSR data set”). The latter images and their manual segmentations were provided by the center for Morphometric Analysis at Massachusetts General Hospital and are available at http://www.cma.mgh.harvard.edu/ibsr/(IBSR version 2.0).

The BrainWeb data set is based on digital phantoms that were made from twenty healthy adults [21], [22], [23], [24]. The images are T1-weighted simulated data with the following parameters: spoiled FLASH sequence, TR = 22 ms, TE = 9.2 ms, flip angle  = 30°, 1 mm isotropic voxel size (3% noise, 0% intensity-inhomogeneity). The corresponding discrete anatomical models consist of an integer value at each voxel that represents the tissue which contributes most to that voxel. We created binary gray matter masks of each of the discrete models and used these masks as ground truth for the corresponding simulated images. The gray matter signal-to-noise ratio in this data set ranged from 47 to 59 (*M* = 53, *SD* = 3.1; see Table 1).

**Table 1:** Image quality parameters of the BrainWeb data set.

	SNR		
Data set	White matter	Gray matter	CNR
BrainWeb data set			
Image 4	59.13	44.48	14.65
Image 5	59.08	46.74	12.34
Image 6	56.80	45.72	11.08
Image 18	50.97	40.80	10.17
Image 20	54.75	43.67	11.08
Image 38	56.09	45.97	10.12
Image 41	50.28	39.95	10.33
Image 42	50.72	40.65	10.07
Image 43	50.26	39.62	10.64
Image 44	53.17	42.86	10.31
Image 45	54.71	43.78	10.93
Image 46	52.02	41.09	10.93
Image 47	50.01	40.10	9.91
Image 48	53.00	42.32	10.68
Image 49	53.06	41.23	11.83
Image 50	53.26	41.89	11.37
Image 51	49.86	38.38	11.48
Image 52	53.33	42.95	10.38
Image 53	47.13	37.35	9.78
Image 54	53.23	41.33	11.90

Note. SNR  =  signal-to-noise ratio; CNR  =  contrast-to-noise ratio.

The IBSR data set consists of high-resolution, T1-weighted volumetric images (resolution at least 1×1×1.5 mm) from 14 male and four female subjects (age: *M* = 38, *SD* = 22.4, including four individuals characterized as juvenile). These images have been reoriented into the Talairach orientation and processed by the Center for Morphometric Analysis biasfield correction routines. Experts segmentations of the principle brain structures include: 3rd ventricle, 4th ventricle, brain stem, and bilaterally: accumbens area, amygdala, anterior amygdala, caudate nucleus, cerebellum cortex, exterior cerebellum, cerebellum white matter, cerebral cortex, exterior cerebral, cerebral white matter, hippocampus, inferior lateral ventricle, lateral ventricle, palladium, putamen, thalamus proper, ventral diencephalon, and vessels. The segmentations are the result of a manually-guided, semi-automatic segmentation technique conducted by a trained expert. Segmentations are provided as structure outlines and as filled volumes. The latter were used in the current study. For the filled volumes, fill codes represents the various structures that were segmented. For the purpose of the current study, the “trinary” representations of the segmentations were used. In these images voxel values have been mapped from the code-to-structure codes into the basic tissue types: background, cerebrospinal fluid, gray matter and white matter. Binary masks for gray matter were created to serve as ground truth for the 18 images. The signal-to-noise-ratio for gray matter in this data set ranged from 16 to 95 (*M* = 47, *SD* = 22.4; see Table 2).

**Table 2:** Image quality parameters of the IBSR data set.

	SNR		
Data set	White matter	Gray matter	CNR
Image 1	47.27	36.28	10.99
Image 2	116.98	94.92	22.06
Image 3	40.01	27.01	13.00
Image 4	23.48	15.86	7.62
Image 5	113.07	80.23	32.84
Image 6	100.85	68.55	32.30
Image 7	69.17	34.42	34.75
Image 8	75.83	43.87	31.96
Image 9	83.92	48.52	35.40
Image 10	51.07	25.02	26.05
Image 11	106.32	58.95	47.37
Image 12	57.38	42.79	14.59
Image 13	39.13	27.52	11.61
Image 14	61.94	40.74	21.20
Image 15	103.84	70.01	33.83
Image 16	111.24	71.60	39.64
Image 17	40.41	26.67	13.74
Image 18	39.43	26.81	12.62

Note. SNR  =  signal-to-noise ratio; CNR  =  contrast-to-noise ratio.

To determine the reliability of different segmentation pathways we acquired ten MRI images of one individual (male, 40 years old; “Single Subject data set”). The first five images were acquired on five different days between October 29^th^ and November 15^th^ 2010. The sixth image was acquired on March 28^th^, 2011. The remaining images were acquired in different sessions on May 19^th^, 2011. The images were acquired on a Siemens Trio (A Tim System, 3 Tesla) with software version Syngo MR B17. Acquisition parameters of the images were as follows: 3D MPRAGE sagittal acquisition of 176 slices (1 mm thickness) with a field of view of 256×256 mm and a matrix of 256×252 resulting in isotropic voxels of 1×1×1 mm^3^; TR = 1900 ms, TE = 2.52 ms, TI = 900 ms, flip angle  = 9 , pixel bandwidth  = 170 Hz, 12 channel head RX-coil, parallel imaging factor 2 (GRAPPA). The signal-to-noise-ratio for gray matter in this data set ranged from 126 to 144 (*M* = 137, *SD* = 7.1; see Table 3).

**Table 3:** Image quality parameters of the Single Subject data set.

	SNR		
Data set	White matter	Gray matter	CNR
Image 1	132.49	86.01	46.48
Image 2	137.74	85.82	51.92
Image 3	128.06	78.71	49.35
Image 4	141.60	89.15	52.45
Image 5	125.76	78.81	46.95
Image 6	143.16	83.24	59.92
Image 7	143.88	84.14	59.74
Image 8	144.12	83.93	60.19
Image 9	131.53	81.86	49.67
Image 10	142.81	64.14	78.67

Note. SNR  =  signal-to-noise ratio; CNR  =  contrast-to-noise ratio.

Additionally, we used the reliability data set provided by the Open Access Series of Imaging Studies (www.oasis-brains.org; “OASIS data set”) [25]. This data set contains twenty subjects scanned on subsequent visits within ninety days. In contrast to Klauschen et al. [20], we only used a subset of the images provided, namely subject numbers 61, 92, 111, 145, 150, 156, 236, 249, 285, and 379 (mean age: 22.7 years, *SD* = 4.7). We chose these particular subjects, because they were scanned twice within twelve days at maximum (*M* = 4, *SD* = 3.8). That way, we ensured that the two scans were maximally similar. The signal-to-noise ratio in gray matter for this data set ranged from 18 to 32 (*M* = 25, *SD* = 4.2; see Table 4).

**Table 4:** Image quality parameters of the OASIS data set.

	SNR		
Data set	White matter	Gray matter	CNR
Image 61/1	17.83	9.30	8.53
Image 62/2	22.44	12.06	10.38
Image 92/1	18.71	10.23	8.48
Image 92/2	19.53	10.71	8.82
Image 111/1	26.10	12.84	13.26
Image 111/2	22.62	10.00	12.62
Image 145/1	21.13	9.10	12.03
Image 145/2	22.92	10.45	12.47
Image 150/1	28.05	13.80	14.25
Image 150/2	28.08	14.39	13.69
Image 156/1	29.91	15.30	14.61
Image 156/2	28.22	14.15	14.07
Image 236/1	29.09	14.28	14.81
Image 236/2	27.96	15.80	12.16
Image 249/1	27.06	13.35	13.71
Image 249/2	27.25	13.09	14.16
Image 285/1	22.05	11.48	10.57
Image 285/2	18.99	9.90	9.09
Image 379/1	25.89	13.17	12.72
Image 379/2	32.27	15.79	16.48

Note. SNR  =  signal-to-noise ratio; CNR  =  contrast-to-noise ratio.

Our study primarily used simulated data and data publicly available. The single subject images were scans that were obtained in the context of continuous quality management at the scanner facility of the Department of Psychiatry and Psychotherapy, Philipps-University Marburg. The images were obtained from J. S., who made the data available for our study.

### Algorithms

We used two preprocessing steps in our analyses: intensity correction and skull-stripping. For intensity correction we used the nonparametric nonuniform intensity normalization (N3) algorithm [26] and for skull-stripping (i) the “watershed” (WS) algorithm of FreeSurfer [27] and the BET algorithm of FSL (version 2.1) [28].

For gray matter segmentation we used (1) “Segment” (provided by SPM8 (r4290): www.fil.ion.ucl.ac.uk/spm/software/spm8/), (2) “New Segment” (provided by SPM8), (3) “VBM8” (provided by the Voxel-based morphometry toolbox for SPM8 (r413): http://dbm.neuro.uni-jena.de/vbm/), (4) “FAST” (version 4.1, provided by FSL (version 4.1.6): www.rmrib.ox.ac.uk/fsl/index.html), and (5) “FreeSurfer” (provided by FreeSurfer (version 4.5.0): (http://surfer.nmr.mgh.harvard.edu/fswiki/FreeSurferWiki).

*Segment* performs segmentation, bias correction and normalization in one step (cf. “Unified Segmentation”) [29]. The underlying generative model includes a correction for intensity non-uniformity and is estimated for a maximum a posteriori solution. *New Segment*, currently work in progress, is an extension of the unified segmentation approach that uses an improved registration model and an extended set of tissue probability maps [30]. The *VBM8* segmentation algorithm uses a maximum a posteriori technique, together with a partial volume estimation and two denoising methods [31]. Additionally, this algorithm integrates the DARTEL normalization [32]. *FAST* uses a hidden Markov random field model and an associated Expectation-Maximization algorithm. The algorithm also corrects for intensity non-uniformities [33]. *FreeSurfer* is a set of tools for the analysis of structural and functional brain imaging data. In its processing stream it allows for subcortical segmentation and cortical parcellation based on prior cortical modeling and a Gaussian classifier atlas [34].

### Study Design and Implementation

**Figure 1:**
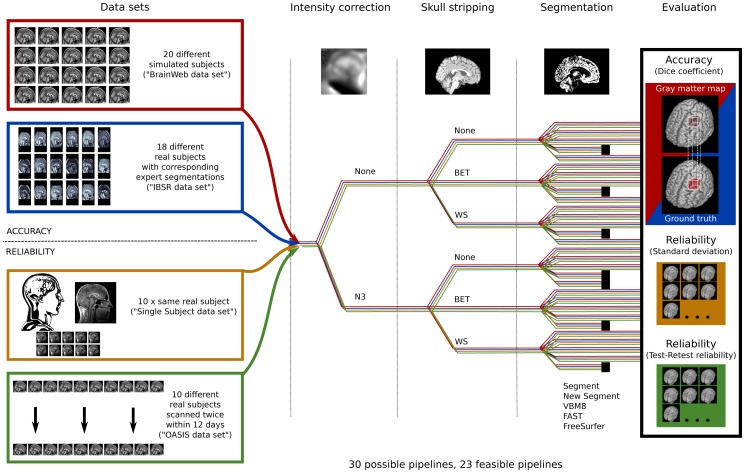
Overview of the study design. In total we processed fifty data sets: (i) twenty simulated brains of the Simulated Brain Database with different anatomical models (“BrainWeb data set”), (ii) 18 different real subjects with corresponding expert segmentations (“IBSR data set”), (iii) ten T1-weighted scans of the same individual (“Single Subject data set”), and (iv) ten pairs of images of subjects who were scanned twice within a maximum of twelve days (“OASIS data set”). We created in total thirty segmentation pathways where each consisted of: An intensity non-uniformity correction preprocessing step (consisting of no intensity correction or *N3*), a skull-stripping preprocessing step (consisting of no skull-stripping, *BET*, or *WS*), and the segmentation of gray matter (via *Segment*, *New Segment*, *VBM8*, *FAST*, or *FreeSurfer*). Once created, we determined that 23 of the total constructed segmentation pathways were feasible for evaluation and these were investigated in the analysis (infeasible pathways are represented with a dot as end marker). To determine the accuracy of the different segmentation pathways we calculated the Dice coefficient for the gray matter maps and corresponding ground truth images for the twenty simulated brains and the IBSR data set. We tested the reliability of the segmentation pathways by (i) determining the variability in terms of standard deviation and coefficient of variation with respect to gray matter volume on the Single Subject data images, and (ii) by calculating the test-retest reliability with respect to gray matter volume for the OASIS data set.

Figure 1 depicts the study design. By combining the algorithms mentioned above for intensity correction (2 possibilities: no intensity correction, *N3*), skull-stripping (3 possibilities: no skull-stripping, *BET*, *WS*), and segmentation (5 possibilities: *Segment*, *New Segment*, *VBM8*, *FAST*, *FreeSurfer*) we created thirty gray matter segmentation pathways in total. Seven of the pathways were not considered further in our study because we regarded these pathways as not relevant in practice. As *FreeSurfer’s* default segmentation procedure already includes *N3* intensity correction and *WS* skull-stripping, combinations with any of the preprocessing steps would have been redundant. In the case of *FAST*, only segmentation pathways that included a skull-stripping step were feasible, because the algorithm assumes brain-extracted data. Thus, we retained a testable pathway total of 23 different processing pathways. We processed each data set with each pathway, resulting in 1564 total calculations of gray matter maps.

Intensity correction was implemented with FreeSurfer’s *mri_nu_correct.mni* program. Skull-stripping was implemented using FSL’s *bet* and FreeSurfer’s *mri_watershed* program with no additional parameters selected except those specifying the input and output volume. Segmentation via *Segment* and *New Segment* was implemented in the batch tool of SPM8 with standard parameters. For *VBM8*, we also used the batch tool of SPM8; here, we used the standard parameters except that we explicitly specified that the output should be saved in native space. For the segmentation with *FreeSurfer* we used the *recon-all –all* command line command. As *FreeSurfer* does not provide a gray matter map right away, we created gray matter masks from the results of the subcortical segmentations and the cortical parcellations and combined these two masks to get the desired gray matter map.

### Evaluation

To quantify the accuracy of gray matter segmentations, we used the Dice coefficient (*DC*) [35], a similarity measure related to the Jaccard index. The *DC* is commonly used to determine accuracy of segmentation methods in neuroimaging settings [36], [37], [38] and is defined as the size of the union of the segmentation result and the ground truth: *DC* = *2TP*/((*FP* + *TP*) + (*TP* + *FN*)), that is, the set of True Positives (*TP*) is divided by the average size of the segmentation result (False Positives (*FP*) + True Positives (*TP*)) and the ground truth (True Positives (*TP*) + False Negatives (*FN*)). A *DC* of 0 indicates no overlap; a value of 1 indicates perfect agreement. Using the *DC*, we evaluated the accuracy of the standard implementations of *Segment*, *New Segment*, *VBM8*, *FAST*, and *FreeSurfer*. With regard to the BrainWeb data set, we resliced the gray matter maps produced by the segmentation pathways to the corresponding ground truth images with a trilinear interpolation. Next, we compared the resliced gray matter maps (binarization threshold: *p*>0.5) and the corresponding ground truth images voxel-wise to calculate the *DC*. With respect to the IBSR data set, segmentation results could be directly compared to the corresponding ground truth images, because original T1 images and ground truth images had the same resolution. Only in case of FreeSurfer, gray matter maps were again resliced to fit the resolution of the corresponding ground truth images. In addition to the *DC*, for each of the five standard segmentation algorithms, we determined the average false positive rate (*f_p_*; cf. specificity) and the average false negative rate (*f_n_*; cf. sensitivity). Moreover, to examine the impact of the choice of the binarization threshold, we also evaluated the gray matter maps using *p*>0.10 and *p*>0.90.

To assess the reliability of the five standard segmentation algorithms, we initially used the Single Subject data set. We calculated the variability in segmented gray matter volumes in terms of the standard deviation in mm^3^ and in terms of the coefficient of variation *c_v_*, which is defined as the ratio of the standard deviation to the mean. Next, we calculated the reliability coefficient *r* for the segmented gray matter volumes measured for the OASIS data set. For this data set, we also computed the average deviation in volume (in %) between the first and second scan.

Finally, to determine which processing factor had the largest impact on segmentation accuracy, we computed separate univariate, three-way analyses of variance (ANOVAs) with according pairwise comparisons for the BrainWeb data set and the IBSR data set. In these analyses, *DC* was the dependent variable and Intensity Correction, Skull-Stripping, and Segmentation were the respective factors for repeated measures. To create a balanced design for statistical analysis, we excluded all pathways that used *FreeSurfer* for segmentation and all pathways that did not use any skull-stripping. Thus, we computed 2 (Intensity correction: none, *N3*) ×2 (Skull-stripping: *BET*, *WS*) × 4 (Segmentation: *Segment*, *New Segment*, *VBM8*, *FAST*) Greenhouse-Geisser corrected ANOVAs with repeated measures on all factors. To further determine which of all five segmentation algorithms tested achieved the highest accuracy, we additionally computed one-way ANOVAs for the factor Segmentation (Segmentation: *Segment*, *New Segment*, *VBM8*, *FAST*, *FreeSurfer*) separately for the BrainWeb and the IBSR data set. Likewise, we computed a one-way ANOVA for the factor Skull-Stripping (Skull-Stripping: none, *BET*, *WS*) to test whether this preprocessing step actually increased or decreased segmentation accuracy in comparison to no prior brain extraction.

## Results

### Accuracy

**Figure 2:**
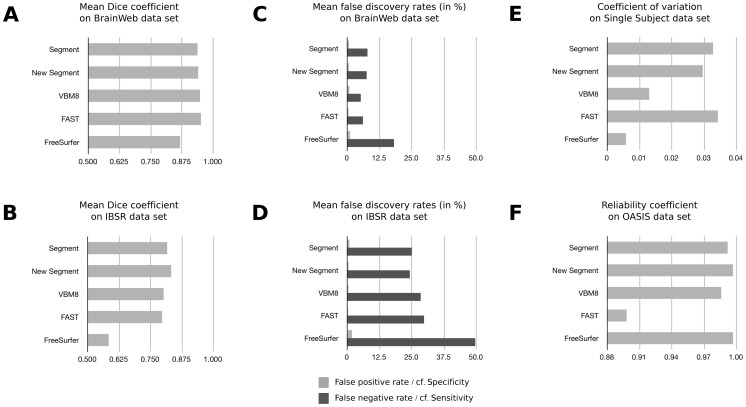
Overview of the results for the five segmentation algorithms in default mode. Depicted is the mean Dice coefficient that was reached by each of the five standard segmentation algorithms in its default mode on the BrainWeb images (Panel A) and on the IBSR data set (Panel B). Panel C and D summarize the average false positive rate f_p_ and the average false negative rate f_n_ for each segmentation algorithm on the Brain Web data set (Panel C) and on the IBSR images (Panel D). Panel E shows the coefficient of variation for gray matter volumes detected in the Single Subject data set. For each segmentation algorithm, Panel F depicts the test-retest reliability determined on the OASIS data set.

Panel A of Figure 2 shows that on the BrainWeb data set *FAST*, *VBM8*, *Segment*, and *New Segment* reached an average *DC* greater than 0.93. *FAST* achieved the highest accuracy (*M* = 0.9513, *SD* = 0.0064), while *VBM8* was slightly lower (*M* = 0.9474, *SD* = 0.0067). *FreeSurfer* showed the lowest accuracy (*M* = 0.8679, *SD* = 0.0087). On the IBSR data set, as illustrated in Figure 2, panel B, *New Segment* reached the highest average *DC* (*M* = 0.8326, *SD* = 0.0129). Compared to *New Segment*, *FAST* (*M* = 0.7962, *SD* = 0.0570), *Segment* (*M* = 0.8167, *SD* = 0.0359), and *VBM8* (*M* = 0.8026, *SD* = 0.0270) showed slightly lower accuracy. FreeSurfer again showed the lowest accuracy of all segmentation algorithms (*M* = 0.5838, *SD* = 0.0570).

Figure 2, panel C and D, illustrates that all segmentation algorithms were especially prone to reduced sensitivity, that is, they tended to underestimate gray matter volume (BrainWeb data set: all *f_n_*s >5%; IBSR data set: all f_n_s >20%). At the same time, all segmentation algorithms showed high specificity (BrainWeb data set: all *f_p_*s <1.5%; IBSR data set: all *f_p_*s <2%). On real data (cf. IBSR data set), *NewSegment* demonstrated the highest sensitivity (*f_n_* = 24.3%) and *FreeSurfer* the lowest (*f_n_* = 49.6%).

For *p*>0.10 instead of *p*>0.50 as binarization threshold, all segmentation algorithms yieled similar accurary on the BrainWeb data set (*DC*s ranged from 0.88 to 0.90). For the IBSR data set, however, *Segment*, *New Segment*, *VBM8* and *FAST* yielded comparable results (*DC*s ranged from 0.84 to 0.87), whereas *FreeSurfer* showed significantly decreased accuracy (0.66). For *p*>0.90, on the BrainWeb data set, again all segmentation algorithms demonstrated comparable accuracy (*DC*s ranged from 0.79 to 0.83). On the IBSR data set, however, only *Segment* and *New Segment* showed feasible accuracy (*DC*s 0.70), whereas *VBM8*, *FAST*, and *FreeSurfer* demonstrated poor accuracy (*VBM8*∶0.61; *FAST*: 0.57; *FreeSufer*: 0.48).

### Reliability

As shown in Figure 2, panel E, *FreeSurfer* showed by far the least variability in segmented gray matter volumes calculated for the Single Subject data set (*SD* = 4504 mm^3^, c_v_ = 0.6%). *VBM8* yielded the second most reliable results (SD = 9998 mm^3^, *c_v_* = 1%), whereas *FAST* (*SD* = 26583 mm^3^, *c_v_* = 3%) and *Segment* (*SD* = 26651 mm^3^, *c_v_* = 3%) showed the largest variability in segmented gray matter volumes. The mean segmented gray matter volumes measured by the five standard segmentation algorithms ranged from 731379 mm^3^ (*FreeSurfer*) up to 820202 mm^3^ (*New Segment*). Thus, the maximum discrepancy between the different segmentation algorithms was 11%. With the exception of *FAST*, all segmentation algorithms showed very high test-retest reliability on the OASIS data set (all *rs* >0.97; please see Figure 2, panel F). *FAST*, however, only demonstrated a reliability coefficient of 0.90. The corresponding average volume differences between first and second scan were: *Segment*: 1.2%, *SD* = 1.1; *New Segment*: 0.6%, *SD* = 0.6; *VBM8*∶2.0%, *SD* = 2.2; *FAST*: 3.3%, *SD* = 2.6; *FreeSurfer*: 1.0%, *SD* = 0.7.

### Impact of Preprocessing Versus Choice of Segmentation Algorithm

For the BrainWeb data set, an Intensity Correction × Skull-Stripping × Segmentation ANOVA on *DC*s revealed a main effect of Intensity Correction (*F*(1,19) = 4.85, *p* = .04, *η* ^2^ = .20), a main effect of Skull-Stripping (*F*(1,19) = 101.69, *p*<.001, *η* ^2^ = .84), and a main effect of Segmentation (*F*(3,57) = 135.89, *p*<.001, *η* ^2^ = .88). Additionally, the analysis revealed a significant Skull-Stripping × Segmentation interaction effect (*F*(3,57) = 34.98, *p*<.001, *η* ^2^ = .65). Bonferroni-corrected post-hoc comparisons revealed that only the factor Segmentation produced meaningful differences in segmentation accuracy. *FAST* produced the most accurate segmentations (*M* = .95, *SD* = .002) and *Segment* produced the least accurate segmentations (*M* = .93, *SD* = .002). The interaction effect indicated no additional practically relevant result, A follow-up one-way ANOVA for the factor Segmentation additionally revealed that *FreeSurfer’s* accuracy (*M* = .87, *SD* = 0.002) was significantly lower than the accuracy of all other segmentation algorithms (Bonferroni-corrected post-hoc comparisons: all *p*’s <.001).

For the IBSR data set, an Intensity Correction × Skull-Stripping × Segmentation ANOVA on *DC*s revealed a main effect of Skull-Stripping (*F*(1,17) = 17.80, *p* = .001, *η* ^2^ = .51) and a main effect of Segmentation (*F*(3,51) = 7.07, *p* = .005, *η* ^2^ = .29), Moreover, the analysis revealed an Intensity Correction × Skull-Stripping interaction (*F*(1,17) = 5.51, *p*<.03, *η* ^2^ = .25), an Intensity × Segmentation interaction (*F*(3,51) = 14.11, *p*<.001, *η* ^2^ = .45), and a Skull-Stripping × Segmentation interaction (*F*(3,51) = 7.69, *p* = .004, *η* ^2^ = .31), Bonferroni-corrected post-hoc comparisons revealed that segmentation pathways using *WS* for skull-stripping produced significantly more accurate segmentations (*M* = .82, *SD* = .006) than pathways using *BET* for skull-stripping (*M* = .80, *SD* = .008). A follow-up one-way ANOVA for the factor Skull-Stripping further revealed that neither *BET* (*M* = .80, *SD* = .004), nor *WS* (*M* = .82, *SD* = .003) yielded an improved accuracy compared to no skull-stripping at all (*M* = .81, *SD* = .008; Bonferroni-corrected post-hoc pairwise comparisons: both *p*s >.30). Further, analyses revealed that, on real T1 MRI data, from all segmentation algorithms *New Segment* yielded the most accurate segmentations (*M* = .82, *SD* = .004). *New Segment’s* accuracy was higher than the accuracy of *Segment* (*M* = .79, *SD* = .009), the accuracy of *VBM8* (*M* = .81, *SD* = .006), and better than the accuracy of *FAST* (M = .80, *SD* = .011). Again, a follow-up one-way ANOVA for the factor Segmentation additionally revealed that *FreeSurfer’s* accuracy (*M* = .58, *SD* = .013) was significantly lower than the accuracy of all other segmentation algorithms (Bonferroni-corrected pairwise comparisons: all *p*’s <.001). Both the Intensity Correction × Skull-Stripping interaction and the Intensity Correction × Segmentation interaction indicated no additional practically relevant effect. The Skull-Stripping × Segmentation interaction, however, indicated that only *Segment* and *New Segment* showed higher accuracy when combined with *WS* (*Segment*: *M* = .81, *SD* = .009; *New Segment*: *M* = .83, *SD* = .004) compared to when combined with *BET* (*Segment*: *M* = .78, *SD* = .01; *New Segment*: *M* = .82, *SD* = .005), whereas *VBM8* and *FAST* showed comparable results for *BET* and *WS*.

## Discussion

### Accuracy of Current Segmentation Algorithms

In the current study, the gray matter segmentation algorithms *Segment*, *New Segment*, *VBM8*, and *FAST* achieved very high accuracy on simulated T1-weighted MRI images (all *DC*s>0.93) and good accuracy on real T1-weighted MRI images (all *DC*s >.79), *FreeSurfer*, however, only achieved a mean *DC* of 0.88 on simulated T1 data and a mean *DC* of.58 on real T1 data. In comparable MRI settings, *DC*s commonly range between 0.75 and 0.97 [36], [37], [38], [39], [40]. From a practical point of view, the average *DC*s of *Segment*, *New Segment*, *VBM8*, and *FAST* are closely comparable. Only FreeSurfer’s accuracy must be considered substantially lower in comparison to the other segmentation algorithms (please see below). Our findings are in agreement with the results of Klauschen et al. [20], who demonstrated that *FAST* and *Segment* have a similar level of sensitivity for gray matter on simulated T1 images (Klauschen et al.: *FAST*: 91%, *Segment*: 90%; current study: *FAST*: 94%, *Segment*: 92%). Importantly, however, our results also demonstrate that sensitivity on real T1 images is substantially lower than on simulated data (e.g., *FAST*: 70%, *Segment*: 75%). Our results are also in accordance with Klauschen et al.’s finding that *FreeSurfer* performs substantially worse than other segmentation algorithms (sensitivity Klauschen et al.: 83%; current study: 82% (simulated T1 data), 50% (real T1 data)). Notably, we also reproduced the findings of Klauschen et al. [20] in that all segmentation algorithms underestimate the actual gray matter volume. This suggests that, in terms of accuracy, the latest algorithmic advancements have not improved segmentation accuracy significantly.

### Reliability of Current Segmentation Algorithms

*VBM8* and *FreeSurfer* demonstrated the most reliable results, whereas *FAST* showed highly variable results. In terms of test-retest reliability, all segmentation algorithms showed almost perfect agreement in segmented gray matter volume. However, the test-retest reliability coefficient for *FAST* was *r* = 0.90, equal to an average deviation of 4% in segmented gray matter volume between the first and second scan. All other segmentation algorithms demonstrated a reliability coefficient of at least 0.99. This finding suggests that, of all tested segmentation algorithms, FAST is most sensitive to varying image quality. *FreeSurfer* and *VBM8*, on the other hand, were the least sensitive to noise factors introduced by different scan sessions. Nevertheless, *VBM8* and *FreeSurfer* still showed an average volume difference between the first and second scan of 2%, or 1% respectively. Taken together, these findings have two important implications: (1) Despite high test-retest reliability, segmentation pathways might still show considerable variations in segmented gray matter volume when several scans of the same subject are segmented. Thus, in accordance with the conclusions of Klauschen et al. [20], our findings further suggest that even segmentation algorithms, which are considered both very accurate and very reliable, still introduce a “segmenter-factor” of up to 3%. This factor has to be considered when morphometric studies of the brain are planned and particularly when results of such studies are interpreted. (2) The fact that we observed pronounced differences in mean segmented gray matter volumes between the different segmentation algorithms strongly emphasizes that findings of segmentation studies that used different segmentation algorithms or different segmentation procedures respectively are not easily comparable.

### Tests on Real Versus Tests on Simulated MRI Images

To our knowledge, our study is the first to investigate segmentation accuracy on real T1-weighted MRI images. Results obtained from simulated images are always limited in their generalized application because simulated images cannot capture the full complexity of real MRI images. Nevertheless, the use of simulated images provides a feasible way of evaluating accuracy, because perfect ground truth images exist in this case. However, as can be seen for example in the case of *FAST*, it is not sufficient to use simulated MRI images to get an idea of how a segmentation algorithm will perform on real data sets. It is of crucial importance to perform tests on both simulated and real MRI data sets, as one may not know all factors that influence the performance of automated segmentation algorithms beforehand. In the current study, for example in the case of *FAST,* only tests on real data sets revealed that the algorithm is highly sensitive to changes in image quality, and only tests on real T1 images could demonstrate that in practice gray matter sensitivity of segmentation algorithms may be up to five times smaller than suggested by evaluations on simulated T1 images. Likewise, Klauschen et al. [20] used simulated data sets of the same subject with variable image quality. In their analysis *FreeSurfer* showed the largest variability in segmented gray matter volume while *FAST* demonstrated a variability that was significantly lower by comparison. Notably, in our study, *FreeSurfer* showed practically no variability whereas *FAST* showed the highest variability on the Single Subject data set. This suggests that even using simulated MRI images with varying image quality cannot replace systematic evaluation of automated segmentation algorithms on real data sets.

### The Impact of Processing Steps

In our study, intensity correction and skull-stripping algorithms applied prior to gray matter segmentation had no impact on later segmentation accuracy that would be of practical relevance. Similarly, Clark et al. [17] found no pronounced differences in segmentation reliability due to intensity correction on their single subject data set. Klauschen et al. [20] also reported no significant influence of skull-stripping algorithms on segmented gray matter volume. Fein et al. [18] reported increased sensitivity in gray matter segmentation for skull-stripped images. However, their results were obtained from SPM2, whose segmentation algorithm suffered from an inaccurate normalization to a T1 brain template and associated problems with the accurate extraction of the brain. Since the implementation of the Unified Segmentation approach [29] these issues obviously do not exist any longer, as can be seen from the fact that *Segment’s* accuracy did not profit from skull-stripping. Acosta-Cabronero et al. [19] demonstrated that skull-stripping may improve SPM5’s segmentation accuracy. However, they used skull-stripping *prior* to intensity correction and furthermore applied algorithms with customized parameters. Because of this, it is difficult to directly compare their results with the results of the current study.

Our results indicate that in particular *Segment* is sensitive to the type of skull-stripping applied. More importantly, however, our results suggest that skull-stripping, may actually decrease segmentation accuracy. This effect might be due to an inaccurate skull-stripping process that cuts out parts of the brain or leaves parts of the skull in the image. These shortcomings could of course be corrected by manual editing. However, in our study, we explicitly wanted to concentrate on fully automated procedures that may be chosen by the average user. Therefore we must conclude that skull-stripping (*BET*, *WS*), in general, should not be used prior to segmentation. The exception is, of course, the processing stream of FSL, where no skull-stripping prior to segmentation produces no feasible segmentation results. In this case, *BET* should be used.

### Performance of FreeSurfer

The results of our analysis of *FreeSurfer’s* accuracy and reliability have to be interpreted cautiously. *FreeSurfer*, in contrast to all other segmentation algorithms reported here, segments and reports gray matter volumes of structures as a whole. All other segmentation algorithms segment a 3D T1 MRI image voxel-wise into tissue classes. In *FreeSurfer*, the definition of the thalamus, for example, extends into the lateral thalamic nuclei, which have a rather heterogeneous tissue composition. The structure labeled as “thalamus” by *FreeSurfer’s* segmentation algorithm may therefore contain both white matter and gray matter. This mechanism may be the reason for *FreeSurfer’s* decreased segmentation accuracy and its high reliability. As *FreeSurfer* labels structures as a whole, the segmentation algorithm is not very sensitive to changes in image quality or noise, which in other algorithms, may lead to misclassifications of single voxels within structures. However, the high reliability caused by this mechanism may become problematic as it is accompanied by low accuracy. Thus, researchers need to decide first, whether they want to focus on identifying cerebral and subcortical structures or gray matter tissue (please see also [41]).

### Limitations and Outlook

The results of the current study have their own limitations. We systematically tested a considerable number of different factors, which may influence segmentation quality, and examined two major measures of segmentation quality, namely accuracy and reliability. However, it is inappropriate to generalize towards every possible scan setting from only the results of our study. Most importantly, this study focused on data processing and was not designed to test technical factors during data acquisition, such as type of coil, impact of parallel imaging, acquisition protocol, or field strength. Future studies may also address regional differences in segmentation accuracy between different segmentation algorithms. Clark et al. [17] implicitly made the first attempt in this direction by calculating gray matter volumes of the major lobes of the brain separately, instead of comparing total gray matter volumes. The focus of further investigations should be to determine which brain segmentation algorithm is most accurate for which region of the brain, most importantly, which segmentation algorithm is best suited for the segmentation of cortical areas, and which algorithm provides the most accurate results for subcortical areas.

### Conclusions

Our findings address crucial factors that influence the quality of gray matter segmentation. Additionally, our results provide guidance in designing state-of-the-art segmentation pathways optimized for individual software settings. Our study emphasizes that comparisons of the results of morphological studies using different segmentation algorithms should be made with great caution. In conclusion, our results suggest that researchers must be aware of the fact that the choice of the segmentation pathway used in a morphometric investigation can easily introduce a “segmenter-effect” on the order of 2–3% variability in segmented gray matter volume. Researchers therefore need to optimize their scanning and processing procedure with respect to their individual settings. Before performing a study, the accuracy and reliability of a specific segmentation pathway has to be adequately determined to enable correct interpretation of the results.
